# Moonlighting functions of the NRZ (mammalian Dsl1) complex

**DOI:** 10.3389/fcell.2014.00025

**Published:** 2014-06-11

**Authors:** Mitsuo Tagaya, Kohei Arasaki, Hiroki Inoue, Hana Kimura

**Affiliations:** Department of Molecular Life Sciences, School of Life Sciences, Tokyo University of Pharmacy and Life SciencesHachioji, Japan

**Keywords:** autophagy, CATCHR tether complex, cell cycle, endoplasmic reticulum, mRNA decay, NAG, RINT1, ZW10

## Abstract

The yeast Dsl1 complex, which comprises Dsl1, Tip20, and Sec39/Dsl3, has been shown to participate, as a vesicle-tethering complex, in retrograde trafficking from the Golgi apparatus to the endoplasmic reticulum. Its metazoan counterpart NRZ complex, which comprises NAG, RINT1, and ZW10, is also involved in Golgi-to-ER retrograde transport, but each component of the complex has diverse cellular functions including endosome-to-Golgi transport, cytokinesis, cell cycle checkpoint, autophagy, and mRNA decay. In this review, we summarize the current knowledge of the metazoan NRZ complex and discuss the “moonlighting” functions and intercorrelation of their subunits.

## Introduction

The unicellular organisms *Escherichia coli* and yeast *Saccharomyces cerevisiae* have roughly 4300 and 6600 genes, respectively, whereas the multicellular organism human contains 22,000–26,000 genes. One may feel that the number of the human genes is too small, given the complex architecture and function of human being. The limited number of genes in multicellular organisms may be due to a strong selective pressure; cells might have evolved not to increase the number of genes because more energy and time are necessary to carry extra genes and accurately transcribe and translate them. To prevent the increase in gene number, multicellular organisms might adopt a strategy to reuse certain proteins for very different cellular processes. Jeffery (1999) coined the term “moonlighting protein” to describe proteins with multiple roles. The list of moonlighting proteins continued to expand and now includes components implicated in membrane trafficking (reviewed by Copley, 2012; Royle, 2013).

The Dsl1 complex in yeast comprises Dsl1 (VanRheenen et al., 2001), Tip20 (Sweet and Pelham, 1993), and Sec39/Dsl3 (Mnaimneh et al., 2004; Kraynack et al., 2005). This complex is a member of the Complex Associated with Tethering Containing Helical Rods (CATCHR) family (Yu and Hughson, 2010) and has been implicated in tethering of Golgi-derived transport vesicles with the endoplasmic reticulum (ER) (Andag et al., 2001; Reilly et al., 2001; Andag and Schmitt, 2003; Zink et al., 2009; Diefenbacher et al., 2011). In 2004, using an immunoaffinity method we isolated from rat liver membranes a large complex containing syntaxin 18, an ER-associated soluble *N*-ethylmaleimide-sensitive factor attachment protein receptor (SNARE) implicated in membrane fusion (Hatsuzawa et al., 2000), and found that the complex includes ZW10 and RINT1 (Hirose et al., 2004). Several lines of evidence suggest that ZW10 and RINT1 are the mammalian counterparts of yeast Dsl1 and Tip20, respectively, although the amino acid sequence identities between the yeast and mammalian proteins are very low (~14%). Intriguingly, both ZW10 and RINT1 had been discovered as cell cycle checkpoint proteins (Williams et al., 1992; Xiao et al., 2001). Nowadays, the mammalian Dsl1 complex is known to participate not only in membrane trafficking and cell cycle but also in other cellular processes including autophagy. In this review, we will focus on the moonlighting functions of the mammalian Dsl1complex, alternatively called the NRZ complex. Regarding the detailed structural and functional features of the Dsl1 complex and other CATCHR family complexes, we refer readers to some excellent reviews (Bröker et al., 2010; Brown and Pfeffer, 2010; Schmitt, 2010; Yu and Hughson, 2010; Bonifacino and Hierro, 2011; Spang, 2012; Hong and Lev, 2014).

## Dsl1 complex: a tether at the ER

In eukaryotic cells, communication between organelles in the secretory and endocytic pathways is mediated by membrane-bound vesicles that transit between organelles. Transport vesicles are formed at the donor compartment, traffic to their destination, lose their coat, and fuse with the acceptor compartment. Docking and fusion of transport vesicles with the target membrane involve an initial contact mediated by Rab GTPases and tethering factors, followed by SNARE-catalyzed membrane fusion.

Tethering factors, which not only facilitate long-range interactions between transport vesicles and the acceptor membrane but also coordinate SNARE complex assembly, can be classified into two groups, large proteins with extended coiled-coils and multisubunit protein complexes. The CATCHR complexes are a subfamily of the multisubunit tethering complexes consisting of the Dsl1, COG, exocyst, and GARP complexes (Yu and Hughson, 2010). Despite subtle sequence homology among their subunits, the CATCHR family tethers have strong three-dimensional structural homology.

**Figure 1:**
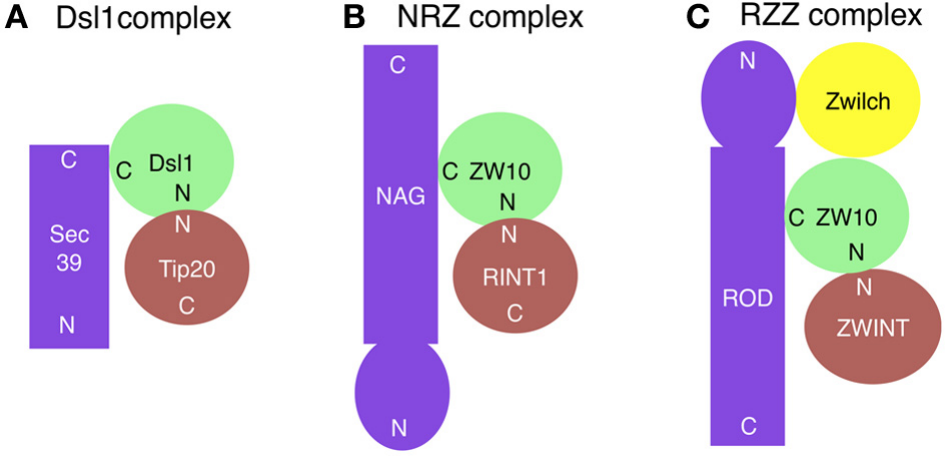
**Subunit compositions of the Dsl1 (A), NRZ (B), and RZZ (C) complexes**. ZWINT is not included in the RZZ complex, but shown here because it may have a role corresponding to Tip20/RINT1 in the Dsl1/NRZ complexes. The ZW10-binding site on ROD has not been mapped. N and C indicate the N- and C-terminal regions, respectively.

**Figure 2:**
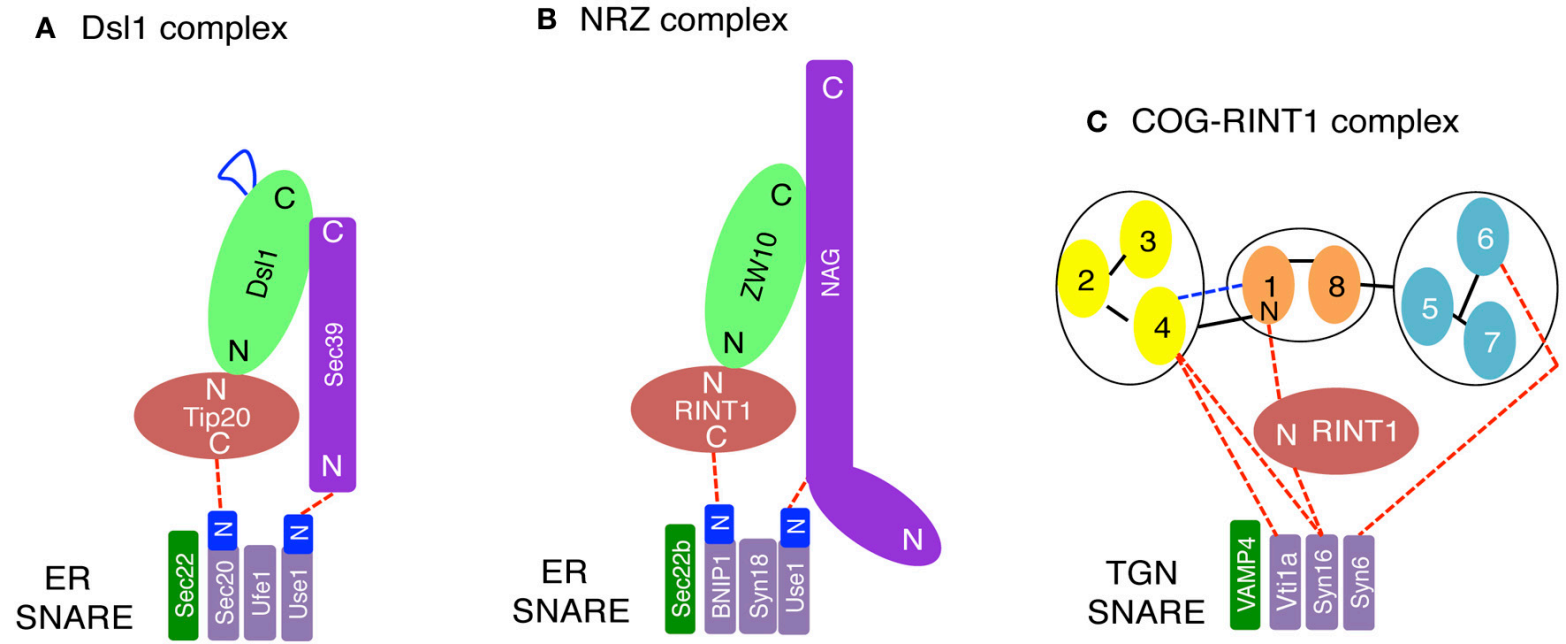
**Interactions between tethers and SNAREs. (A)** On yeast ER membranes, Tip20 and Sec39 bind to the N-terminal regions (N) of Sec20 and Use1/Slt1, respectively. The region of Dsl1 shown in blue represents an acidic region that interacts with COPI components. **(B)** On the mammalian ER, RINT1 and NAG bind to the N-terminal regions of BNIP1 and Use1/p31, respectively. **(C)** On the mammalian TGN, SNAREs binds the COG complex directly or indirectly through RINT1. The COG complex model (Oka et al., 2005; Ungar et al., 2005) has been slightly modified. N and C indicate the N- and C-terminal regions, respectively.

The yeast Dsl1 complex, which comprises three subunits (Dsl1, Tip20, and Sec39/Dsl3) (Figure 1A), has been implicated in tethering of Golgi-derived transport vesicles on the ER (Andag et al., 2001; Reilly et al., 2001; Andag and Schmitt, 2003; Zink et al., 2009; Diefenbacher et al., 2011). Although all these proteins are rich in α-helices, their origins may be different; Dsl1 and Tip20 have a common α-helical fold, thus originating from an ancestral CATCHR protein (Tripathi et al., 2009), whereas Sec39/Dsl3 lacks the shared fold and thus appears to be derived from a non-related protein (Ren et al., 2009). The Dsl1 complex resides on ER membranes and binds to and regulates the assembly of the ER SNAREs Ufe1, Sec20, Use1/Slt1, and Sec22 (Figure 2A) (Kraynack et al., 2005; Ren et al., 2009; Diefenbacher et al., 2011). The Dsl1 complex has been predicted to form a 20-nm-tall tower from the ER surface, which can allow an interaction with COPI-coated vesicles (Ren et al., 2009). The COPI (α- and δ-COPs)-binding sites in Dsl1 have been defined to its central acidic region (Andag and Schmitt, 2003), which is located in the tip of the tower (Ren et al., 2009). An additional role of the Dsl1 complex is to assist uncoating of COPI-coated vesicles tethered on ER membranes. In this role, Dsl1 may block domains in COPI that drive repolymerization and the formation of large COPI aggregates (Zink et al., 2009).

While the roles of the yeast Dsl1 complex have been so far entirely limited to the tethering function on the ER, one study has suggested the involvement of the Dsl1 complex in peroxisome biogenesis. Previously, peroxisomes were recognized as an autonomous organelle, but recent studies have revealed a primary role of the ER in the *de novo* formation of peroxisomes (reviewed by Agrawal and Subramani, 2013). Searching for ER-associated proteins responsible for this role revealed that all Dsl1 complex components are required for peroxisome biogenesis (Perry et al., 2009). It was speculated that Dsl1 complex components may function as a tether for retrograde carriers from peroxisomes (Nagotu et al., 2010) or an anchor to recruit dynein for peroxisome biogenesis (Perry et al., 2009), but the mechanism by which Dsl1 complex subunits regulate peroxisome biogenesis should be elucidated in future studies.

The mammalian counterpart of the Dsl1 complex was identified by us (Hirose et al., 2004; Aoki et al., 2009) and was later called the NRZ complex for its subunit names, NAG (Sec39/Dsl3), RINT1 (Tip20), and ZW10 (Dsl1) (Civril et al., 2010) (Figure 1B and Table 1). Like the yeast Dsl1 complex (Sweet and Pelham, 1993; Kraynack et al., 2005; Ren et al., 2009; Tripathi et al., 2009; Diefenbacher et al., 2011), the NRZ complex is associated with the ER SNAREs syntaxin 18 (Ufe1), BNIP1 (Sec20), p31 (Use1/Slt1), and Sec22b (Sec22) (Figure 2B) (Hatsuzawa et al., 2000; Hirose et al., 2004; Nakajima et al., 2004; Aoki et al., 2009; Uemura et al., 2009). The yeast Dsl1 complex interacts with another SNARE, Ykt6 (Meiringer et al., 2011), but it is not clear whether or not the NRZ complex binds to the mammalian ortholog of this SNARE. The mechanism underlying SNARE complex assembly appears to be somewhat different between mammals and yeast. In mammals, the assembly of the ER SNAREs occurs in the absence of RINT1 (Arasaki et al., 2006), whereas yeast Tip20 plays a pivotal role in ER SNARE complex assembly (Kraynack et al., 2005; Diefenbacher et al., 2011). Moreover, the binding of Sec22b to syntaxin 18 in mammals creates high-affinity binding sites for BNIP1 and p31 (Aoki et al., 2008), whereas yeast Ufe1, Sec20, and Use1/Slt1 form a stable complex in the absence of Sec22 (Kraynack et al., 2005).

**Table 1:** **Features of subunits of the human NRZ and RZZ complexes**.

**Proteins**	**Binding regions with partners**	**Structural features**
ZW10 (779 aa^*^)	1–170 aa: RINT1, dynamitin 1–82 aa: ZWINT	Putative coiled-coil regions^**^ (13–38 aa, 53–83 aa, 107–130 aa, 356–383 aa, 616–636 aa)
RINT1 (792 aa)	1–264 aa: ZW10, COG1	Putative coiled-coil regions (60–92 aa, 101–126 aa, 163–183 aa)
	151–256 aa: UVRAG	
	358–440 aa: RBL2/p130	
	257–792 aa: Rad50	
NAG (2371 aa)	1036–2371 aa: ZW10-RINT1	β-propeller (1–420 aa)
		Sec39-like (734–1355 aa)
ROD (2209 aa)	1–350 aa: Zwilch	β-propeller (1–390 aa)
		Sec39-like (557–1153 aa)
Zwilch (591 aa)	Not determined	Putative coiled-coil regions (69–89 aa, 290–311 aa)

* *aa, amino acids*.

** *window size = 21*.

The Dsl1 complex is conserved in plants. Screening for *Arabidopsis* mutants that abnormally accumulate the precursors of storage proteins, 2S albumin and 12S globulin, in dry seeds identified a mutant, designated maigo2 (MAG2: At3g47700) (Li et al., 2006). MAG2 is the ortholog of RINT1 (Tip20). Affinity purification identified three MAG2-binding proteins, MIP1 (At2g32900), MIP2 (At5g24350), and MIP3 (At2g42700). MIP1 and MIP2 share sequence homology with ZW10 (Dsl1) and NAG (Sec39/Dsl3), respectively, (Li et al., 2013). MIP3 is a member of the Sec1 family domain-containing proteins, named SCFD2. SCFD2 is present in mammals, and may bind to ZW10 (Hutchins et al., 2010), although our original study failed to detect this protein in the syntaxin 18 immunoprecipitates (Hirose et al., 2004). Instead, we found Sly1/SCFD1 in the precipitates (Hirose et al., 2004), likely due to the direct binding of Sly1/SCFD1 to syntaxin 18 (Yamaguchi et al., 2002). The *Arabidopsis* Dsl1 complex is implicated in abscisic acid-mediated response to abiotic stresses. This response may be related to Dsl1 complex-mediated membrane trafficking between the ER and Golgi (Zhao et al., 2013). There is a homolog of MAG2, named MAG2-like (MAG2L: At1g08400) in *Arabidopsis* (Li et al., 2006), and MAG2L is partially redundant with MAG2 in response to environmental stresses (Zhao et al., 2013).

## RZZ complex: alternate ZW10-containing complex

To preserve genetic information, the genomes of organisms must be accurately replicated and segregated before cell division. In eukaryotes, during mitosis and meiosis, sister-chromatid segregation occurs after all kinetochores form stable bipolar microtubule attachments. If not correctly attached to the spindle, kinetochores activate the spindle assembly checkpoint, leading to the block of cell cycle progression.

ZW10 (Zeste White 10) and ROD (Rough Deal) were discovered as proteins that are required for faithful chromosome segregation in *Drosophila* (Karess and Glover, 1989; Williams et al., 1992). Both proteins were found to be conserved in humans (Starr et al., 1998; Scaërou et al., 2001). At the onset of mitosis, these proteins redistribute from the cytoplasm to kinetochores (Williams et al., 1992; Scaërou et al., 1999) and recruit the core spindle checkpoint proteins Mad1-Mad2 to microtubule-unattached kinetochores (Buffin et al., 2005; Kops et al., 2005; Gassmann et al., 2010). Once all kinetochores become stably attached to the spindle, ZW10 and ROD are transported away from kinetochores by dynein (Howell et al., 2001; Wojcik et al., 2001; Basto et al., 2004). The removal of these proteins from kinetochores acts as a signal for the termination of the spindle checkpoint (Basto et al., 2000; Chan et al., 2000). ZW10 interacts with dynamitin, a subunit of the dynein-dynactin complex (Echeverri et al., 1996), thereby recruiting this motor to kinetochores (Starr et al., 1998). ZW10 and ROD form a large complex of 800 kDa, and immunoaffinity purification identified the third component, Zwilch (Williams et al., 2003), which encouraged Karess (2005) to call this complex RZZ for ROD-ZW10-Zwilch (Figure 1C). The binding of ROD and NAG to ZW10 is mutually exclusive, suggesting that the RZZ and NRZ complexes exist as distinct entities. Structural prediction revealed unexpected similarity between ROD and NAG. They share an N-terminal β propeller followed by an α solenoid, which is a characteristic structure of certain nucleoporins such as Nup133 and vesicle coat subunits such as clathrin heavy chain and α-COP (Civril et al., 2010). In Nup133, the C-terminal α solenoid structure is responsible for the localization at the nuclear pore complex in interphase and at kinetochores in mitotic cells, whereas the N-terminal β propeller domain interacts with CENPF, which can recruit dynein-dynactin via NudE or its homolog NudEL (Stehman et al., 2007; Bolhy et al., 2011). This protein–protein interaction chain (Nup133-CENPF-NudE or NudEL-dynein-dynactin) is reminiscent of the RZZ-mediated recruitment of dynein-dynactin to kinetochores. ROD binds to Zwilch through its N-terminal β propeller domain (Civril et al., 2010), as does Nup133 to CENPF.

Zwilch and RINT1, both of which are ZW10 binding partners, do not share any structural similarity, suggesting that Zwilch is not a descendant of the ancestral CATCHR protein (Civril et al., 2010). Moreover, in contrast to RINT1, Zwilch and ROD are not well conserved in eukaryotes (Schmitt, 2010), raising the possibility that the RZZ complex was generated after the occurrence of the NRZ complex accompanied by the acquisition of an additional function by ZW10. This is consistent with the view that the RZZ complex is a fairly recent add-on to the core spindle checkpoint complex (Vleugel et al., 2012). ROD and Zwilch always co-occur in Opisthokonta, and their co-occurrence correlates well with the presence of flagella and centrioles in Opisthokonta (Schmitt, 2010). The co-appearance of ROD and Zwilch might allow microtubules and their motor dynein to function in the termination of spindle checkpoint.

In *Drosophila* there is no NAG ortholog, and ROD may function as a substituent for NAG in membrane trafficking (Wainman et al., 2012). In *Drosophila* spermatocytes, ROD and ZW10 accumulate at the Golgi apparatus, as well as the ER in the case of ZW10, and these proteins are required for Golgi stack integrity (Wainman et al., 2012). The presence of ZW10 on both ER and Golgi membranes has also been reported in COS-7 (Varma et al., 2006) and rodent cells (Arasaki et al., 2007). Depletion of *Drosophila* RINT1, as well as mutations in the *ZW10* and *ROD* genes, causes apparent alteration in Golgi morphology and reduces the number of Golgi stacks. In contrast, Zwilch is not associated with membranes and its mutation does not affect Golgi structure (Wainman et al., 2012). ZW10 and RINT1 are necessary for another membrane trafficking-related event, meiotic cytokinesis in spermatocytes, but not for mitotic one (Williams et al., 1992). In ZW10 or RINT1 mutant spermatocytes, regular central spindles and actomyosin rings can assemble, but furrow ingression halts prematurely due to a defective plasma membrane addition. On the other hand, neither ROD nor Zwilch is required for cytokinesis (Wainman et al., 2012).

## Multiple functions of NRZ subunits

### ZW10

During interphase, in addition to the retrograde transport from the Golgi to the ER, ZW10 may participate in dynein-mediated movement of endosomes and lysosomes (Varma et al., 2006). It is not clear whether ZW10 functions in the anterograde transport from the ER to the Golgi, which is mediated by dynein-dynactin that moves toward the minus-end of microtubules (Presley et al., 1997). One study showed that depletion of ZW10 causes the release of dynein from membranes (Varma et al., 2006). Moreover, ZW10 moves along microtubules toward their minus-end and accumulate at the centrosome in interphase and at kinetochores in mitosis, when the interaction of ZW10 with dynein-dynactin is stabilized (Inoue et al., 2008; Famulski et al., 2011). However, a recent study strongly suggests that ZW10 acts as a tether rather than a dynein anchor (Majeed et al., 2014). It should be clarified in future studies how ZW10 participates in tethering despite the lack of an acidic region responsible for the interaction with COPI-coated vesicles in yeast Dsl1 (Andag and Schmitt, 2003; Schmitt, 2010).

At the onset of mitosis, microtubules switch their role from the transport of membranous structures, such as Golgi, endosomes, lysosomes, and transport vesicles, to that of non-membranous chromosomes as well as of kinetochore proteins. ZW10, ROD, and perhaps many other kinetochore proteins are supposed to move along microtubules from the cytosol to the nucleus (Williams et al., 1992; Scaërou et al., 1999). The finding that Golgi disassembly is required for entry into mitosis (Sütterlin et al., 2002) might be related to this phenomenon. Presence of the Golgi structure might be an “obstacle” for massive protein transport along microtubules to the nucleus, and its loss may allow cytosolic and ER- and Golgi-associated kinetochore proteins to enter the nucleus just before and during nuclear envelope breakdown.

During mitosis, ZW10 interacts with several kinetochore-associated proteins, such as ZWINT (Starr et al., 2000), C19orf25 (Kops et al., 2005), and PIASy (Ryu and Azuma, 2010). ZWINT was first identified by yeast two-hybrid screen as a ZW10-binding protein (Starr et al., 2000). This 43-kDa protein, predicted to be largely coiled-coil, is recruited to kinetochores in early prophase, before the earliest detection of ZW10, and remains there until mid-anaphase (Starr et al., 2000; Wang et al., 2004). ZWINT and RINT1 may have equivalent functions in different ZW10-containing complexes. Like RINT1 (Inoue et al., 2008), ZWINT interacts with the N-terminal region of ZW10 and helps the recruitment of the ZW10-containing complex to the target architecture, kinetochores (Starr et al., 2000; Wang et al., 2004; Famulski et al., 2008).

ZWINT may also play a role in membrane trafficking in interphase cells. It interacts with membrane trafficking proteins such as Rab3c, SNAP25, SNAP29, and GM130 (Lee et al., 2002; van Vlijmen et al., 2008; Hutchins et al., 2010). Both Rab3c and SNAP25 bind to the same region of ZWINT (amino acids 79–179), which includes a putative coiled-coil region (van Vlijmen et al., 2008). Rat ZWINT, called SIP30 for SNAP-25 interacting protein of 30 kDa, is upregulated in the central nervous system in response to neuropathic pain (Zhang et al., 2009), and is required for neuropathic pain-evoked aversion (Han et al., 2014).

A very recent study has revealed an unexpected role for ZWINT. ZWINT has been shown to directly interact with Beclin1 whose loss results in a significant reduction of the outer kinetochore proteins including ZW10, leading to a defect in chromosome congression (Frémont et al., 2013). Beclin1 is the mammalian ortholog of yeast ATG6/VPS30 and a typical moonlighting protein participating not only in autophagy, but also in endocytosis, apoptosis, and inflammation (reviewed by Salminen et al., 2013).

### RINT1

RINT1 was originally discovered as a Rad50-interacting protein required for G_2_/M cell cycle checkpoint control (Xiao et al., 2001). Depletion of RINT1 causes not only partial Golgi fragmentation (Arasaki et al., 2006; Sun et al., 2007), but also defects in mitosis, including the formation of multiple spindle poles and frequent chromosome missegregation (Lin et al., 2007). RINT1 heterozygous mice quite often developed multiple tumors due to haploinsufficiency, suggesting that RINT1 serves as a tumor suppressor (Lin et al., 2007). However, a recent integrative functional genomics study has validated that *RINT1* is a glioblastoma oncogene that can confer tumorigenicity to primary nontransformed murine astrocytes *in vivo* (Quayle et al., 2012). An additional role for RINT1 is to interact with Rb-related 2 (RBL2/p130) and control telomere length (Kong et al., 2006). Yeast Tip20 may function in the nucleus because crystal-like structures are formed in the nucleus of Tip20 mutants (Spang, 2012).

Recent studies by us revealed an unanticipated involvement of RINT1 in endosome-to-*trans*-Golgi network (TGN) trafficking. Deletion of RINT1 causes a more severe dispersal of TGN proteins than that of *cis*-Golgi proteins due to a defect in endosome-to-TGN trafficking (Arasaki et al., 2013). In this context, RINT1 does not work with ZW10, but cooperates with the COG complex (Figure 2C), another member of the CATCHR family complexes (Yu and Hughson, 2010). RINT1 binds to COG1 through its N-terminal 264-amino acid region, which is also a ZW10-binding region (Hirose et al., 2004; Arasaki et al., 2006). This fact may explain why the COG-containing RINT1 complex is distinct from the NRZ complex. RINT1 binds to the SNARE (C-terminal coiled-coil) domain of syntaxin 16, a SNARE implicated in endosome-to-TGN trafficking (Mallard et al., 2002), and regulates SNARE complex assembly presumably at the TGN (Arasaki et al., 2013). The region of COG1 responsible for the association with RINT1 is the N-terminal 93-amino acid region containing a putative coiled-coil region, which has been identified as a VPS51-like domain in the Pfam database. VPS51 is a member of the GARP complex consisting of VPS51/Ang2, VPS52, VPS53, and VPS54 (Pérez-Victoria et al., 2010). Although RINT1 binds to the VPS51-like domain of COG1, it does not bind to Vps51 or other GARP subunits (Arasaki et al., 2013). The COG and GARP complexes are also involved in endosome-to-TGN trafficking through the interaction with TGN SNAREs (Pérez-Victoria and Bonifacino, 2009; Pérez-Victoria et al., 2010; Laufman et al., 2011, 2013; Willett et al., 2013). The requirement of many different tethers for endosome-to-TGN trafficking likely reflects the presence of multiple sources of vesicles that traffic to the TGN. Moreover, the participation of RINT1 in this pathway may reflect adaptation to the demand for more diverse transport routes from endosomes to the TGN in mammals compared with those in a unicellular organism, yeast.

**Figure 3:**
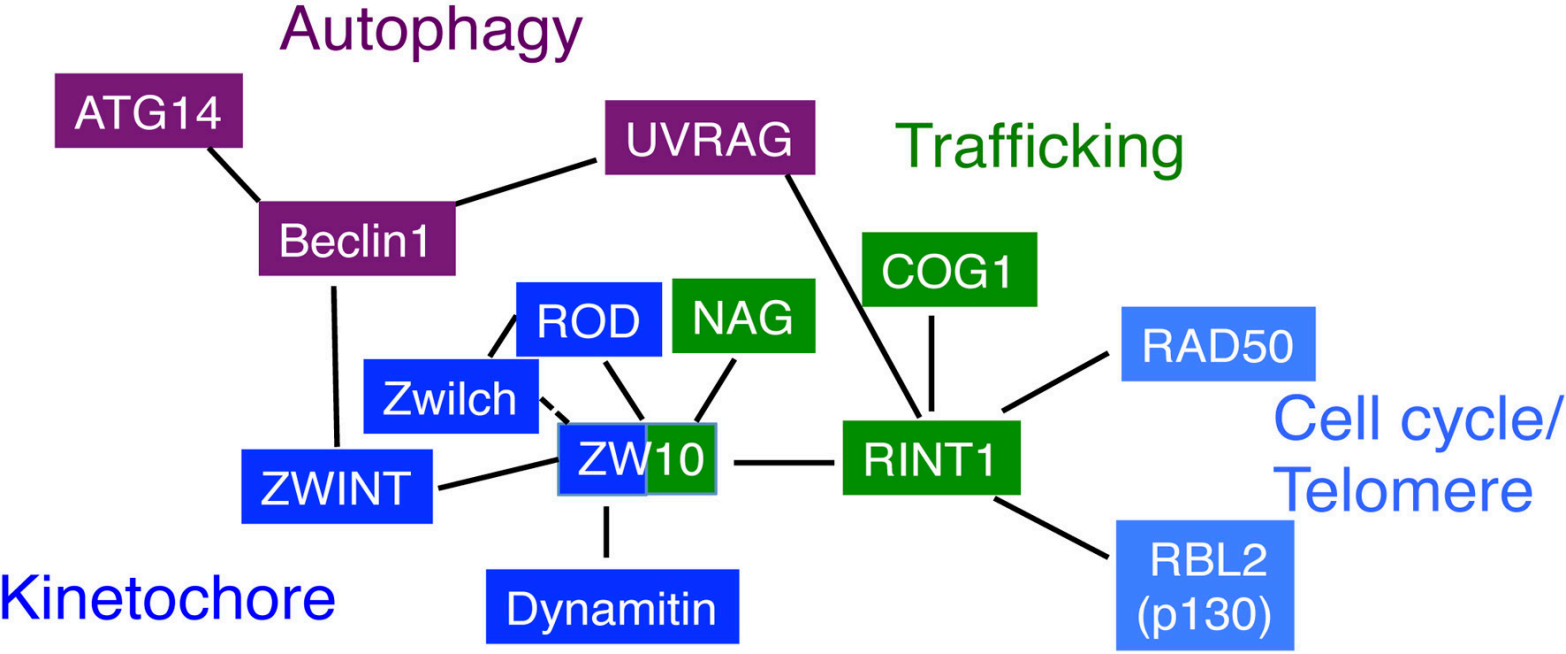
**Correlation diagram of NRZ complex subunits**. ZW10 is the center for the interactions. Through its N-terminal, putative coiled-coil region, it interacts with RINT1 for membrane trafficking (Hirose et al., 2004; Inoue et al., 2008), dynamitin for recruiting the dynein-dynactin complex (Starr et al., 1998; Inoue et al., 2008), ZWINT for the association with kinetochores (Wang et al., 2004; Kops et al., 2005). The C-terminal region of ZW10 likely interacts with NAG and ROD (Kraynack et al., 2005; Aoki et al., 2009; Civril et al., 2010). The interaction of Zwilch with ZW10 is not tight in the absence of ROD (Civril et al., 2010). On the other hand, RINT1 interacts with COG1 through its N-terminal, putative coiled-coil region (Arasaki et al., 2013). This interaction mode may be a common for the interactions of CATCHR family members. The N-terminal region is also responsible for the interaction with UVRAG, but in this case, RINT1 keeps the binding to ZW10 (He et al., 2013). The RINT1 interacts with RAD50 and RBL2/p130. UVRAG interacts with RINT1 under basal conditions and, upon starvation, dissociates from RINT1 and interacts with Beclin1. Of note is that Beclin1 during mitosis interacts with ZWINT, a ZW10 partner.

Autophagy is a process to engulf, degrade, and recycle cytoplasmic contents, and is required for cell survival in response to starvation. Recent work by Liang and colleagues (He et al., 2013) revealed a connection between RINT1 and autophagy. UV-radiation resistance-associated gene (UVRAG) interacts with Beclin1 through its coiled-coil region, which in turn activates phosphatidylinositol 3-kinase for autophagy (Liang et al., 2006; Itakura et al., 2008). It also plays a role in endocytosis under basal conditions (Liang et al., 2008). He et al. (2013) discovered that, in fed cells, UVRAG is associated with RINT1 and participates in tethering of COPI vesicles in cooperation with the NRZ complex. Upon starvation, it dissociates from RINT1 and binds to Beclin1, which in turn promotes ATG9 translocation from the Golgi to the autophagosome biogenesis site (He et al., 2013). It should be noted that Beclin1 interacts with ZWINT (Frémont et al., 2013), suggesting a connection between membrane trafficking (RINT1), kinetochore function (ZWINT), and autophagy (Beclin1-UVRAG) (Figure 3).

### NAG

The neuroblastoma-amplified gene (NAG), alternatively called neuroblastoma-amplified sequence (NBAS), was first identified as a gene co-amplified with the *MYCN* gene in neuroblastoma (Wimmer et al., 1999). This amplification occurred likely because a 2.8 Mb non-fragile region containing the *MYCN* and *NAG* genes flanks the *FRA2C* region, a common fragile site of chromosomes (Blumrich et al., 2011). The association of NAG with tumor progression and prognosis remains controversial (Scott et al., 2003; Weber et al., 2004; Kaneko et al., 2007). Although NAG is well conserved in eukaryotes, the molecular size is very different between fungi and other eukaryotes (Schmitt, 2010). In many fungi, the molecular size of the predicted NAG orthologs, such as yeast Sec39/Dsl3, is about 80–100 kDa, whereas in other eukaryotes, the size is almost 2 to 3-fold larger than fungal proteins because of the presence of a long N-terminal extension comprising a β propeller domain and a long C-terminal extension, in addition to the central Sec39-like domain (about 13% homology between human and yeast).

The larger size of metazoan NAGs may allow them to have additional functions. Indeed, NAG has been reported to participate in nonsense-mediated mRNA decay (NMD) (Longman et al., 2007, 2013; Anastasaki et al., 2011). The NMD pathway is an elaborate surveillance mechanism that triggers the degradation of mRNAs containing premature termination codons (PTCs) and also regulates ~10% of naturally occurring transcripts (reviewed by Chang et al., 2007). A genome-wide RNA interference screen in *Caenorhabditis elegans* identified NAG and a member of the DEAD/DEAH-box helicases, DHX34, as factors responsible for the NMD pathway independent of core NMD factors (SMG1-7) (Longman et al., 2007). This pathway is conserved in zebrafish and human (Anastasaki et al., 2011), and NAG and DHX34, like core NMD factors, co-regulate a significant proportion of genes (Longman et al., 2013). Given that DEAD/DEAH-box helicases are commonly involved in many aspects of RNA metabolism including transcription, pre-mRNA splicing, translation, and mRNA decay (reviewed by Fuller-Pace, 2006), the involvement of DHX34 in the NMD pathway is understandable. However, the mechanism underlying NAG-mediated NMD is totally unknown. Interestingly, a mutation in the NAG gene has been shown to be a cause for the hereditary short stature syndrome (SOPH syndrome) in Yakuts, who live in the far east of the Russian Federation (Maksimova et al., 2010). This autosomal recessive disorder is associated with optic nerve atrophy and Pelger-Huët anomaly, the latter of which is characterized by an abnormal nuclear shape in neutrophil granulocytes (Maksimova et al., 2010). These symptoms may be explained by the fact that NAG is involved in the NMD pathway and ER function. As the outer nuclear membrane is contiguous with the ER, disruption of the ER structure may affect the nuclear morphology.

*Nicotiana benthamiana* NAG comprises 2409 amino acids, thus can be classified to non-fungal NAGs, and its silencing causes ER stress and cell death (Lee et al., 2013). This phenotype may be relevant to that observed in MAG2 mutants (Zhao et al., 2013).

## Conclusions and perspectives

Figure 3 shows the summary of protein–protein interactions between NRZ components. Many, but not all, interactions are mediated through putative coiled-coil regions. As described above, the NRZ complex, as well as the Dsl1complex, consists of two distinct structural units. ZW10 and RINT are derived from the CATCHR family ancestor, and NAG is from a different one. Because a CATCHR family member RINT1 interacts with another CATCHR family member COG1 (Arasaki et al., 2013), it is tempting to speculate that RINT1 orthologs in other eukaryotes may also interact with CATCHR family members. Indeed, a comprehensive analysis of protein–protein interactions in yeast demonstrated that Tip20 can interact with COG4 (Uetz et al., 2000). Like RINT1, ZW10, and its orthologs may also interact with CATCHR family members. Given that SCFD1 and SCFD2 bind to the mammalian COG4 (Laufman et al., 2009) and plant MAG2 (Li et al., 2013), respectively, it is worth examining the interactions between CATCHR family members and Sly1 family (SCFD) proteins. The currently available data suggest that the role of RINT1 in autophagy is passive; it anchors UVRAG until autophagy induction. However, as the ER is a major source of autophagosome formation (reviewed by Lamb et al., 2013), RINT1 and other NRZ complex subunits may have more active roles in autophagy.

Schmitt (2010) has proposed that the ancestor of animals and fungi probably expressed both sets of ZW10-interacting proteins (NAG- RINT1 and ROD-Zwilch). Rod and Zwilch encoding genes have been assumed to be lost independently in different phylogenetic branches, and Sec39 likely lost the N-terminal β-propeller domain in parallel with the acquisition of the acidic COPI-interacting region by Dsl1. We would like to suggest another possibility. The ancestor of NAG/Sec39 acquired a β-propeller domain with a C-terminal extension for yet unrevealed functions, and the occurrence of Zwilch might facilitate the appearance of ROD by gene duplication. In this context, it is important to identify proteins that interact with the β-propeller domain and/or C-terminal extension of NAG. Moreover, elucidation of the molecular mechanism by which NAG mediates the NMD pathway may provide a clue to the reason for the association/cooperation of CATCHR proteins with NAG.

### Conflict of interest statement

The authors declare that the research was conducted in the absence of any commercial or financial relationships that could be construed as a potential conflict of interest.
